# Effect of climate change on bud phenology of young aspen plants (*Populus tremula*. L)

**DOI:** 10.1002/ece3.3352

**Published:** 2017-09-01

**Authors:** Unnikrishnan Sivadasan, Tendry Randriamanana, Cao Chenhao, Virpi Virjamo, Line Nybakken, Riitta Julkunen‐Tiitto

**Affiliations:** ^1^ Natural Products Research Laboratories Department of Environmental and Biological Sciences University of Eastern Finland Joensuu Finland; ^2^ Faculty of Environmental Sciences and Natural Resource Management CERAD Norwegian University of Life Sciences Ås Norway

**Keywords:** bud break, bud set, phenology, *Populus tremula*, temperature, UVA, UVB

## Abstract

Boreal tree species are excellent tools for studying tolerance to climate change. Bud phenology is a trait, which is highly sensitive to environmental fluctuations and thus useful for climate change investigations. However, experimental studies of bud phenology under simulated climate change outdoors are deficient. We conducted a multifactorial field experiment with single (T, UVA, UVB) and combined treatments (UVA+T, UVB+T) of elevated temperature (T, +2°C) and ultraviolet‐B radiation (+30% UVB) in order to examine their impact on both male and female genotypes of aspen (*Populus tremula* L.). This study focuses on the effect of the treatments in years 2 and 3 after planting (2013, 2014) and follows how bud phenology is adapting in year 4 (2015), when the treatments were discontinued. Moreover, the effect of bud removal was recorded. We found that elevated temperature played a key role in delaying bud set and forcing bud break in intact individuals, as well as slightly delaying bud break in bud‐removed individuals. UVB delayed the bud break in bud‐removed males. In addition, both UVA and UVB interacted with temperature in year 3 and even in year 4, when the treatments were off, but only in male individuals. Axillary bud removal forced both bud break and bud set under combined treatments (UVA+T, UVB+T) and delayed both under individual treatments (T, UVB). In conclusion, male aspens were more responsive to the treatments than females and that effect of elevated temperature and UV radiation on bud set and bud break of aspen is not disappearing over 4‐year study period.

## 1 INTRODUCTION

Global average surface temperatures have increased by 0.85°C over the period from 1880 to 2012 (Intergovernmental Panel on Climate Change (IPCC), 2014). As Earth's temperature rises, it becomes warmer earlier in the spring and stays warmer later into the fall at any location (Karl, Nicholls, & Gregory, 1997). This global warming could result in numerous changes in the nature. For instance, increase in temperature can affect the reproductive and dispersal potential of insects (Ayres & Lombardero, 2000), and future climatic effects on plant phenology may change the availability of forage for dependent animal species (Mysterud, Yoccoz, Langvatn, Pettorelli, & Stenseth, 2008). Mismatches in timing between dependent animal species and plants may result in species outbreaks and/or extinctions. Plants are generally finely tuned to the seasonality of their environment. The autumn phenology of trees was traditionally thought to be mainly controlled by day length (Wareing, 1956), and spring phenology by temperature (Zohner, Benito, Svenning, & Renner, 2016). Recently conducted studies, however, have shown that rising temperatures influences the flushing dates of northern tree species (e.g., Chung et al., 2013; Ibáñez et al., 2010; Menzel, 2000). There are also signs of autumn phenology being affected by increase in temperature (Westergaard & Eriksen 1997, Kalcsits, Salim, & Tanino, 2009; Tanino, Kalcsits, Silim, Kendall, & Gray, 2010; Rohde, Bastien, & Boerjan, 2011; Hänninen & Tanino, 2011; Way, 2011). The commencement of autumn leaf senescence and dormancy is based on a combination of numerous developmental and environmental signals (Cooke, Eriksson, & Junttila, 2012). Climate warming can accelerate the growth cessation in many tree species, cultivars, and ecotypes (Tanino et al., 2010). In some cases, the growth cessation occurs as a combination of low night temperatures and photoperiod. However, in some northern ecotypes of *Picea abies*,* Salix pentandra,* and *Betula pubescens* whose dormancy induction is insensitive to photoperiod, the autumn growth cessation is delayed due to the temperature increase resulting in longer growing seasons (Hänninen & Tanino, 2011).

The effects of climate factors on phenology have mostly been studied in growth chambers or in common gardens (e.g., Beuker, 1994; Burley, 1966; Hannerz, 1994; Hänninen, 1990; Hurme, Repo, Savolainen, & Pääkkönen, 1997; Leinonen, 1996; Myking & Heide, 1995; Oleksyn, Tjoelker, & Reich, 1998) while there are few outdoor studies with a specific experimental setup. The growth conditions used in almost all experiments indoors are temporarily and spatially less variable than those in natural environments (Frenkel, Jankanpaa, Moen, & Jansson, 2008). The temperatures are usually constant or at the best varying between diurnal values, while the light intensity is generally lower and with a spectral composition differing quite significantly from that of the sun (Leonidopoulos, 2000; Young, McMahon, Rajapakse, & Decoteau, 1994). On the other hand, while in common garden experiments, phenology can be studied in natural light and temperature conditions, the local versus foreign effect cannot be tested rigorously (Allendorf & Lundquist, 2003). Thus, field experiments with experimentally modulated light and temperature supplementation are needed in order to gain a better understanding of the effects of climate factors on phenology.

Growth and dormancy cycles in plants are also controlled by light quality (Olsen & Lee, 2011). Blue, red, and far‐red light influences the growth, dormancy, and bud formation of many plant species (e.g., Campbell et al., 2008; Mølmann, Junttila, Johnsen, & Olsen, 2006; Olsen, 2010). The effects of the UVA and UVB regions of the solar spectrum, however, have not been widely investigated. Most of the research on UVB and plants has concentrated on the effect of UVB as a stress factor. However, there is increasing evidence that UVB may also function as an environmental signal (Jenkins, 2009; Zlatev, Lidon, & Kaimakanova, 2012). Strømme et al. (2015) found that UVB accelerates bud set and bud break in European aspen (*Populus tremula*. L) plants after one growing season, while Sivadasan, Randriamanana, Julkunen‐Tiitto, and Nybakken (2015) found variations in secondary metabolites and bud size in male and female *Salix myrsinifolia* buds under UV radiation.

*Populus tremula* is a diecious deciduous tree that is widely distributed throughout Europe and Asia. *Populus* species are of great ecological importance, as a large number of organisms, including several endangered species, are found in association with these trees (e.g., Lindroth, 2008). They are, therefore, monitored in several national and international phenology networks. *Populus* species are widely used as model organisms among woody plants in experimental botany (e.g., Bradshaw, Ceulemans, Davis, & Stettler, 2000; DiFazio, Slavov, & Joshi, 2011). Numerous studies on climate change are conducted with different *Populus* species as its circumboreal range largely overlaps with areas where drastic climate change is predicted to occur (IPCC 2014). *Populus* species had been found to show sex‐specific responses under different climatic stress factors (Li et al., 2014; Randriamanana et al., 2014). For example, Strømme et al. (2015) have shown that in young *P. tremula* plantlets, elevated temperature delayed bud set and forced bud break in one growing season old seedlings. However, in their studies, under combined UVB+T treatment, bud set was forced in both males and females while bud break was delayed only in males. Moreover, at low temperatures, females of *P. cathayana* showed earlier growth cessation and more chilling injuries in the chloroplast ultrastructure, cellular membranes, and leaf morphology compared to males (Zhang, Jiang, Peng, Korpelainen, & Li, 2011). Also, variations between sexes as to the magnitude of morphological, physiological, and biochemical traits have been documented under drought and elevated temperature in *P. cathayana* (Xu, Peng, Wu, Korpelainen, & Li, 2008). Males of *P. cathayana* having higher basal diameter, leaf nitrogen, and lower concentration of abscisic acid and UV‐absorbing compounds and exhibited greater resistance under enhanced UVB than did females (Xu et al., 2010).

This study used the same experimental setup as Strømme et al. (2015), except that we investigated the subsequent 2‐year effects of climate change on *P. tremula* bud phenology, and the potential carry‐over effects for 1 year after the treatments were discontinued. In addition, the effect of axillary bud removal on the timing of bud set and bud break was tested. We hypothesized that (i) enhanced temperature will delay bud set and force bud break, while UVB will force bud set, and the responses will be mitigated by the experimental years due to acclimation, (ii) the effects of enhanced temperature on bud break will be sustained over the following season, even when the treatments are discontinued, (ii) bud removal will change the growing period due to resource restrictions, and (iv) males and females vary in their responses to the treatments.

## 2 MATERIALS AND METHODS

### 2.1 Plant materials

The aspen plantlets used in this experiment originated from Eastern and Southern Finland, as presented in Randriamanana, Nissinen, Moilanen, Nybakken, and Julkunen‐Tiitto (2015). In 2012, when the field experiment started, they were micropropagated from buds of six male and six female aspen trees, about 30–40 years old. Each genotype was collected from the following locations: Kaavi 62^°^43′N, 28^°^42′E, Liperi 62^°^41′N, 29^°^33′E, Loppi 60^°^43′N, 24^°^27′E, Pieksämäki 62^°^18′N, 27^°^07′E, Polvijärvi 62^°^52′N, 29^°^19′E and 62^°^49′N, 29^°^20′E, and Kontiolahti 62^°^38′N, 29^°^41′E. Geographical distance was maintained in order to have the largest possible variation among the genotypes. The micropropagation was performed on a woody plant medium with 8.5 g/LAgar and 5 mg/L indole butyric acid. Fluorescent tubes (Gro‐Lux F36W, Havells Sylvania, Germany) of photon flux density 70‐μmol m^−2^ s^−1^ at 400–750 nm were used to provide light at 23 ± 0.1^°^C temperature and 18 hr photoperiod in vitro.

When transferred to the greenhouse on 2 May 2012 for acclimatization, the plantlets were potted up with 70% commercial peat (Kekkilä Oy, Lapinneva, Finland) and 30% vermiculite (AO Vermipu Oy, Lapinjärvi, Finland). The relative air humidity was set at 70%. High‐pressure sodium lamps (GE Lighting, Cleveland, OH, USA) of 400 W were used for enriching the light conditions. The temperature was set to 20 ± 3^°^C, and the photoperiod was 18 hr. Due to the additional heat production from the lamps, the temperatures fluctuated between 20 and 23^°^C, depending on the time of day. The plantlets were transferred to the field site in Joensuu, Finland (62^°^60′N, 29^°^75′E), on 7 June 2012 and planted in soil on 11 June. We followed the same individuals for bud phenology as in Strømme et al. (2015). Some mortality occurred during the study period occurred due to *Venturia* shoot blight, and also as a result of some mechanical and herbivore damages. All the plants in the experimental field were scored for bud break and bud set. As a consequence of *Venturia* infections on the apical meristems, numerous buds remained dead or fell off during scoring, and only the plants that successfully in completed their growth stages were taken into consideration for the data and statistical analyses. The reduction in the number of individuals in 2014 was due to severe *Venturia* infection, while there was an increase in the number in 2015 owing to new growth during the subsequent spring season. The number of plants recorded for bud set in 2013 was 289 females and 319 males and in 2014, 203 females and 197 males. The number of plants recorded for bud break in 2014 was 261 females and 291 males and in 2015, 413 females and 405 males.

### 2.2 Experimental setup

The experimental setup included 36 plots in a 6 × 6 matrix, as explained in detail by Nybakken, Hörkkä, and Julkunen‐Tiitto (2012). The plants within each plot received one of the following six treatments or treatment combinations: enhanced temperature (T), enhanced ultraviolet‐B radiation (UVB), ultraviolet‐A radiation (UVA), UVB+T, UVA+T, and control with ambient temperature and UV radiation (C). The enhanced levels of T and UVB were continuously regulated to increases of +2^°^C and 30%, respectively. A 10 cm layer of 0.8% limed mineral soil was added to each plot. A distance of 3 m was kept between the plots in all directions, and adjustable aluminum frames (1.5 × 2.0 m) holding the lamps and heaters above the plots were bolted to metallic posts. A metal net fence of 1.5 m was constructed around the experimental field in order to prevent the intrusion of large mammals, and a protective metal sheet was implanted 60 cm into the soil, reaching 60 cm above the soil level, to prevent vole intrusion.

To each aluminum frame, six 40 W UV fluorescent lamps (1.2 m long, UVB‐313, Q‐Panel Co., Cleveland, OH, USA) were appended, following a cosine distribution (Björn, 1990). The emission spectrum was measured with an Optronic OL‐756 portable UV‐VIS spectroradiometer (Optronic Laboratories, Orlando, FL, USA), while cellulose diacetate filters were wrapped around each lamp to debilitate radiation below 290 nm in the UVB treatment plots. The UVB plots received some additional UVA emitted by the UVB tubes. In six plots, the UV tubes were wrapped with polyester film in order to remove UVB, so that only the levels of UVA were achieved. The purpose of the UVA treatment was to provide control for UVB, as there were certain levels of UVA in the UVB plots. In the ambient UV plots, we hung un‐energized lamps in order to procure the same level of shading as in the enhanced plots. Two infrared (IR) heaters (CIR 110, FRICO, Partille, Sweden) were bolted along the middle axis of the aluminum frames for continuous temperature enhancement. In the ambient temperature plots, IR radiators were replaced with wooden boards in order to attain the same shading levels. The filters were changed every 3 weeks, and the frames were lifted every third week in order to maintain a 60 cm distance between the highest shoot tip and the radiators/UV lamps.

Four Thies Clima sensors (Thies, Göttingen, Germany) were used for measuring UVB radiation. These sensors measured the radiation between 250 and 325 nm, with a peak of 300 nm. Two sensors were placed above the control frames for ambient UVB levels, and two under the frames of UVB enhancement plots for set‐point values. Temperature enhancement modulation was achieved using self‐made linear temperature sensors with four PT1000 probe elements fabricated with four connection cables. The set‐point values were achieved by placing two probe elements above the control frames and two under the temperature enhancement frames. Calculations of set‐point values and control of enhancement of the UV lamps and IR radiators were implemented by a modulator software (IPC100 configuration program and e‐console measuring and data‐saving program, Gantner Instruments GmbH, Darmstadt, Germany). The whole system was in operation between 5 June 2013(day 156) and 13 September 2013 (day 256). In 2014, the treatment setup was in operation from 8 May (day 128) 2014 until 28 July (day 209) 2014. In 2015, the treatment system was not in operation at all. The ambient temperature data for the years 2013, 2014, and 2015 were procured from the meteorological weather station at Linnunlahti, Joensuu (<200 m away from the experimental field). The photoperiod data were obtained from the sunrise‐and‐sunset.com website for Joensuu, Finland. As the photoperiod curves for 2013, 2014, and 2015 were overlapping and indistinguishable, only the 2013 photoperiod is included in the figure (Figure 4).

### 2.3 Axillary bud removal

In summer 2014 (2nd July) (day 183), three axillary buds from four lateral shoots were removed from one individual of every clone in each plot (total 410 individuals). In autumn 2014 (20th October) (day 293), another three buds were removed from the same individual in order to see whether the bud removal had any effect on bud set and bud break for the coming growing season.

### 2.4 Scoring the bud set and bud break stages

**Figure 1:**
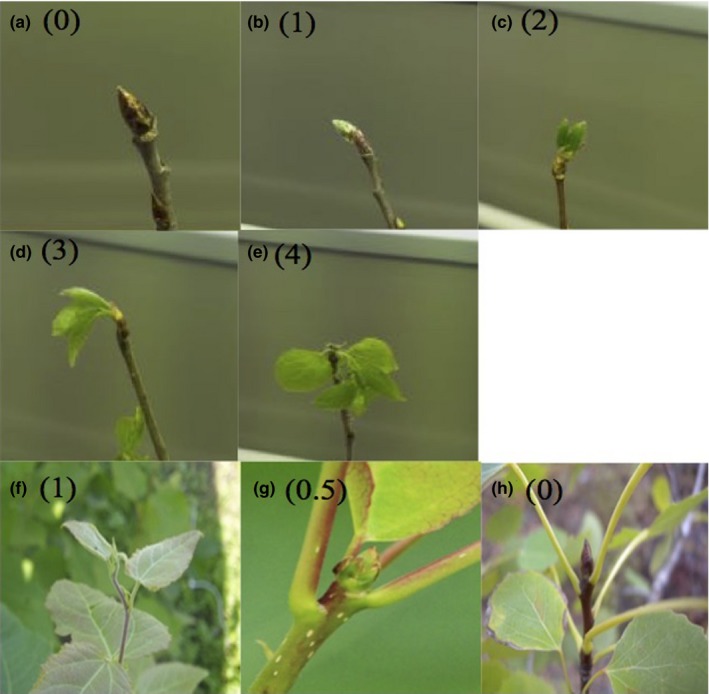
The bud break stages used for scoring the spring phenology were (a–e), a (0)—a closed brown bud, b (1)—closed bud with protruding green leaf tips, c (2)—green leaf tips out of the bud with leaf bases hidden, d (3)—broken bud with at least one petiole and e (4)—unfolded leaf with visible leaf blade and stalk. The bud set stages used for autumn phenology scoring were f–h, f (1)—apices between full, active growth to apices with an open bud, g (0.5)—a closed green bud, and h (0)—a closed brown bud

In 2013, the autumnal bud set was recorded from 20 August (day 232) until 19 October, 2013 (day 292) at 5‐day intervals, while the remaining three registrations were made at 2‐day intervals. In 2014, the registering of bud break took place from 22 April (day 112) to 25 June (day 176). Bud set for autumn 2014 was recorded from 12 August (day 224) to 7 October (day 280). In 2015 bud break scoring started on 4 May (day 124) and ended on 25 June (day 176). The scoring of bud set was based on Rohde et al. (2011), and the defining of bud break stages was based on Fu, Campioli, Deckmyn, and Janssens (2012). The first stage includes apices between full, active growth to apices with an open bud (1), the second stage is a closed green bud (0.5), and the third a brown closed bud (0) (Figure 1). In the case of bud break, the stages were defined as follows: a closed bud (0), closed bud with protruding green leaf tips (1), green leaf emerged from the bud with leaf bases hidden (2), broken bud with at least one visible petiole (3), and an unfolded leaf with visible leaf blade and stalk (4) (Figure 1).

### 2.5 Statistical analysis

The effects on bud break (2014, 2015) and bud set (2013, 2014) were analyzed using the cumulative link mixed model (clmm) in R (R Core Team, 2016) by applying the clmm function in Ordinal package (Christensen, 2015). Two levels of temperature (ambient and enhanced), three levels of UV treatment (ambient, UVA, UVB), two levels of sex (male and female), and the day of year were set as fixed factors. Random factors were plot and clone identities. When more than one individual per clone and experimental plot were recorded, individual was nested within clone and plot. Along with these, interactions between sex, day, temperature, and UV treatments were also analyzed. For the analysis, only the time frame in which there were marked changes in bud stages was considered as follows: for bud break data from 2014—4 May–28 May (days 124–148), 2015 intact individuals—4 May–5 June (124–156), 2015 bud‐removed individuals—4 May–11 June (days 124–156) and in case of bud set data 2013—20 August–7 October (days 232–268), 2014 intact individuals—12 August–25 September (days 224–258) and 2014 bud‐removed individuals—12 August–25 September (days 224–258).

## 3 RESULTS

### 3.1 Bud set in 2013 and 2014

**Figure 2:**
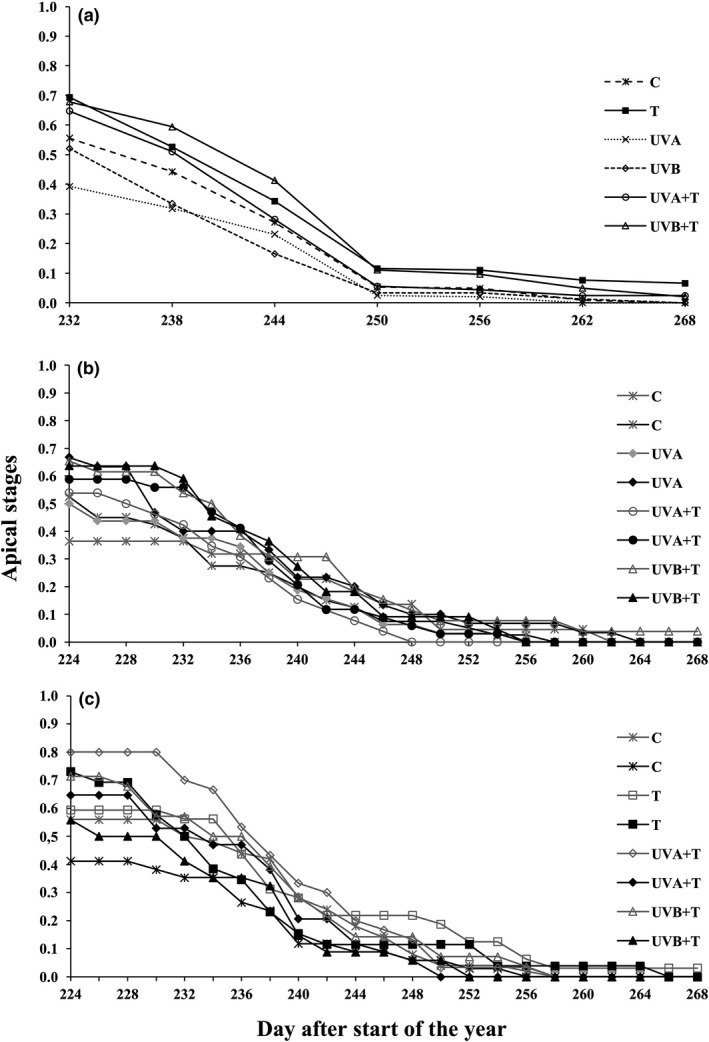
Average score values of apical stages of intact and bud‐removed male and female aspen plantlets during bud set. 2013 bud set (a) (both male and female average score values combined), 2014 bud set (b = intact, c = bud removed) (for intact and bud‐removed individuals, only the treatments having significant effects are included in the graphs along with control treatments and female comparisons in order to avoid the complexity in visualization). (black markers = significant treatments, gray lines = females, T, elevated temperature, UVA, Ultraviolet radiation‐A, UVB, Ultraviolet radiation‐B, UVA+T, Ultraviolet radiation‐A+ elevated temperature, UVB+T, Ultraviolet radiation‐B+ elevated temperature)

During autumn 2013, elevated temperature delayed bud set in both males and females (Figure 2a). In autumn 2014, UVA treatment delayed bud set in males, but the combination of UVA and temperature (UVA+T) forced the buds to set earlier in males than in females (Table 1, Figure 2b). In the same year, removal of axillary buds resulted in delayed bud set in males under elevated temperature, relative to females (Figure 2c). However, after bud removal, the combined treatment UVB+T forced the buds to set earlier in males compared to females. The combination treatment UVA+T also forced the bud break in males, to a smaller extent (Table 1, Figure 2c).

**Table 1:** Parameter estimates, *SE*,* z*‐values, and *p* values for covariates in the cumulative link mixed model run to test the effects of elevated temperature, UVA, and UVB and their combinations on bud set in *Populus tremula* individuals during autumn 2013 and 2014 Bold p values denote the significant treatments and interactions

Fixed effect terms	Coefficient	*SE*	*z*	*p*
Bud set 2013				
T	−1.947	0.492	3.955	**≤.001**
UVA	0.834	0.591	1.411	.158
UVB	0.186	0.592	0.314	.754
Male	−0.170	0.820	−0.208	.835
Bud set 2014				
T	−0.379	0.857	−0.443	.657
UVA	0.129	0.856	0.151	.880
UVB	−0.748	0.799	−0.937	.348
Male	0.601	0.939	0.640	.522
UVA × T	0.603	1.168	0.516	.605
UVB × T	0.855	1.058	0.808	.418
T × Male	−0.264	0.359	−0.737	.461
UVA × Male	−2.137	0.353	−6.050	**≤.001**
UVB × Male	−0.455	0.328	−1.386	.165
UVA ×T × Male	1.534	0.488	3.142	**.001**
UVB × T × Male	0.821	0.498	1.649	.099
Bud set 2014 bud removed				
T	−0.106	0.512	−0.207	.835
UVA	−0.511	0.506	−1.009	.313
UVB	−0.218	0.491	−0.444	.656
Male	1.182	0.830	1.423	.154
UVA × T	0.283	0.704	0.402	.687
UVB × T	−0.437	0.716	−0.611	.541
T × Male	−1.683	0.333	−5.049	**≤.001**
UVA × Male	−0.086	0.287	−0.302	.762
UVB × Male	−0.229	0.288	−0.794	.427
UVA × T × Male	0.893	0.433	2.062	**.039**
UVB × T × Male	2.123	0.454	4.673	**≤.001**

### 3.2 Bud break in 2014 and 2015

**Figure 3:**
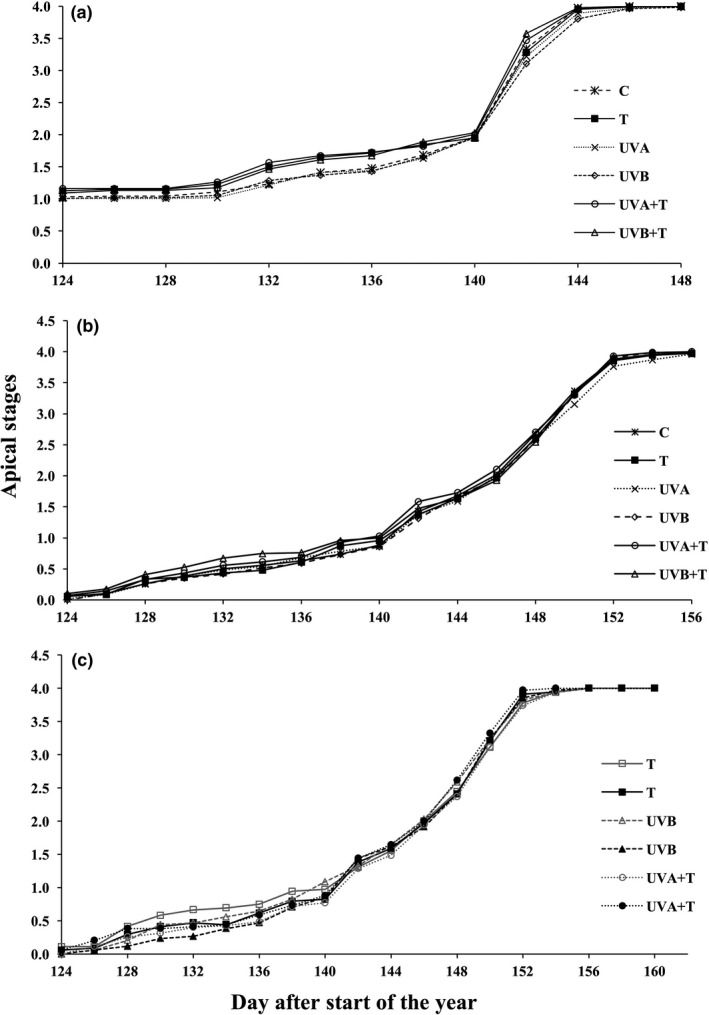
Average score values of apical stages of intact and bud‐removed male and female aspen plantlets during bud break. 2014 bud break (a) (both male and female average score values combined), 2015 bud break (b = intact, c = bud removed) (for intact individuals, male and female average score values are combined and for bud‐removed individuals only the treatments having significant effects are included in the graphs along with their female comparisons in order to avoid the complexity in visualization). (black markers = significant treatments, gray lines = females, T, elevated temperature, UVA, Ultraviolet radiation‐A, UVB, Ultraviolet radiation‐B, UVA+T, Ultraviolet radiation‐A+ elevated temperature, UVB+T, Ultraviolet radiation‐B+ elevated temperature)

Bud break was forced under temperature treatments in both males and females in 2014 by 2 days (Figure 3a) and to a smaller degree in spring 2015 (Figure 3b). The significant negative coefficient of males showed that the female bud break was earlier than males, independent of treatments (Table 2). In 2015, when buds had been removed, elevated T and UVB slightly delayed bud break in males when compared to female clones (Table 2). In contrast, the combination of temperature and UVA (UVA+T) enhanced bud break in males compared to female clones. (Table 2, Figure 3c).

**Table 2:** Parameter estimates, *SE*,* z*‐values, and *p* values in the cumulative link mixed model run to investigate the effects of elevated temperature, UVA, and UVB and their combinations on bud break of *Populus tremula* individuals during spring 2014 and 2015 Bold p values denote the significant treatments and interactions

Fixed effect terms	Coefficient	*SE*	*z*	*p*
Bud break 2014				
T	2.012	0.283	7.106	**≤.001**
UVA	−0.144	0.336	−0.431	.667
UVB	−0.425	0.342	−1.241	.215
Male	−1.209	0.818	−1.478	.139
Bud break 2015				
T	0.330	0.152	2.168	**.030**
UVA	0.068	0.186	0.369	.712
UVB	0.071	0.186	0.384	.700
Male	−0.184	0.057	−3.222	**.001**
Bud break 2015 bud removed				
T	0.576	0.324	1.766	.075
UVA	−0.006	0.327	−0.020	.983
UVB	0.371	0.325	1.140	.254
Male	−0.008	0.318	−0.026	.979
UVA × T	−0.655	0.460	−1.424	.154
UVB × T	0.010	0.458	0.023	.981
T × Male	−0.456	0.205	−2.218	**.026**
UVA × Male	0.125	0.210	0.595	.552
UVB × Male	−0.575	0.207	−2.778	**.005**
UVA ×T × Male	0.779	0.291	2.671	**.007**
UVB × T × Male	0.520	0.288	1.807	.070

## 4 DISCUSSION

In this study, we followed, to our knowledge, for the first time, the effects of temperature and UV on bud set and bud break in woody plant seedlings for three subsequent years. In line with our first hypothesis, and similar to the first year (Strømme et al., 2015), bud set was delayed by elevated temperature during the second year (autumn 2013). This confirms that temperature modifies sensitivity to day length signals at growth cessation and can influence the duration of bud formation in *P. tremula*, as seen earlier both in another outdoor study carried out at different field sites for two seasons with hybrids from *P. nigra, P. trichocarpa*, and *P. deltoides* (Rohde et al., 2011), and in a study conducted in a controlled environment with hybrids from *P. nigra, P. petrowskyana,* and *P. deltoides* (Kalcsits et al., 2009). Likewise, bud removal delayed bud set in males when compared to females. Bud removal can result in an overall reduction in sugar levels; sugars having a cross‐talk with the pathways of phytohormones also causing imbalances between them (Eyles et al., 2013; Gibson, 2005; Little & Wareing, 1981). It is also found that sugars do play an important role in controlling bud dormancy by influencing the phytohormones (Anderson, Chao, & Horvath, 2001; Horvath, Anderson, Chao, & Foley, 2003). High abscisic acid concentration is also associated with bud dormancy (Rinne, Tuominen, & Junttila, 1994), and the removal of buds could lower the concentration of this hormone, leading to a delayed bud set in males under elevated temperature. The bud‐removed individuals in our experimental field showed increased height growth (Sobuj et al. unpublished data), and it has also been found that the increased height growth is genetically associated with delayed bud set in *P. balsamifera* (Riemenschneider, McMahon, & Ostry, 1992).

**Figure 4:**
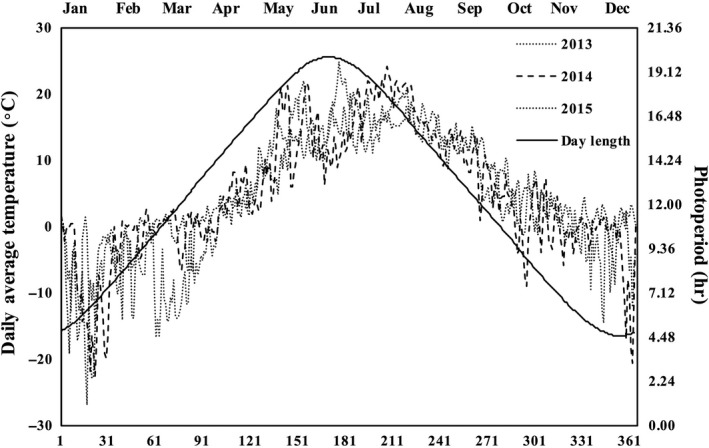
Ambient daily average temperature at the experimental site for the years 2013, 2014, and 2015. The photoperiod (solid line) for the year 2013 is also shown

The spring time temperatures during bud break in 2014 and 2015 were very different (Figure 4). In 2014, several fluctuating warm periods provoked bud opening to the first stage even before recording started. However, bud break was over 8 days earlier in 2015. An effect of enhanced temperature on bud break, as similarly detected by Strømme et al. (2015), was recorded in 2014 and to a small degree in 2015. Earlier studies show that spring events, such as leaf unfolding or needle flush, are particularly sensitive to temperature (Lechowicz, 1995; Sarvas, 1972, 1974). In accordance with our results on the after effect of the treatments in 2015, Fu et al. (2012) found that *Betula pendula, Fagus sylvatica,* and *Quercus robur* were affected by the temperatures from the previous year. It can be speculated that in addition to the carry‐over effect of the temperature treatment, the enhanced bud break in intact individuals in 2015 could have also resulted from the previous year high autumn temperature which might have influenced the subsequent bud burst. Elevated temperature can alter the sugar levels in buds (Pagter, Andersen, & Andersen, 2015), and bud break is associated with low levels of soluble sugars (Lipavská, Svobodová, & Albrechtová, 2001). In this case, the enhanced temperature would have reduced the sugar concentrations, as was also seen in *B. pendula* seedlings (Riikonen et al., 2013), which could be the mechanism behind the temperature‐forced bud break in 2014 and 2015.

Contrary to our hypothesis, UVB did not affect bud break and bud set during the treatment period. In the first growing season (2012), UVB had a forcing effect on the bud break of male clones (Strømme et al. (2015), but the plants may have acclimated to the climatic treatments. We are not aware of any 3‐year prolonging studies of phenology under enhanced UV, but our results are in line with Bassman, Edwards, and Robberecht (2002) from *Pseudotsuga menziesii* after 3 years under supplemental UVB. They found no significant differences in growth, photosynthesis, and UV‐absorbing compounds. Increasing tolerance in plants to UVB over time is partly due to the production of UVB absorbing compounds that can reduce the transmittance of UV photons through leaf tissue (Jansen et al., 1996). Responsiveness to UV dose gradually decreases in leaves as plants age (Kakani, Reddy, Zhao, & Gao, 2004; Klem et al., 2012; Urban, Tuma, Holub, & Marek, 2006), which can also be one reason for the disappearance of the UVB effect after the first year in our experiment.

After removal of axillary buds, males previously exposed to elevated temperature set their buds later than females. However, within plants previously exposed to UVB+T, only females had delayed bud set (autumn 2014) and also had earlier bud break (spring 2015) than males. This gender differences in bud phenology in response to UVB and temperature treatments are difficult to explain. In bud‐removed individuals, UVB might have also caused some fluctuations in the carbohydrate levels during the bud break causing a delay as explained by Lindroth, Hofman, Campbell, McNabb, and Hunt (2000)and Quaggiotti, Trentin, Dalla, and Ghisi (2004). Bud development might be also related to growth processes, which in turn may have been altered by bud removal. In fact, under UVB+T and before bud removal, males had bigger biomass than females (Nissinen et al., unpublished data). Thus, bud removal might have caused females to compensate for their relatively delayed growth when the treatments were discontinued.

According to our hypothesis, the bud phenology of male and female aspens differed in their responses to environmental changes. The males of some species of *Populus* are more growth‐oriented than females (Lloyd & Webb, 1977). In a greenhouse experiment, the males of *P. tremula* were taller had higher shoot biomass and greater leaf area when compared to females (Randriamanana et al., 2014). This may mean that male bud development and entry into the vegetative phase may be faster than in females. In *P. tomentosa*, An et al. (2011) found that during the time of floral budding, the male buds normally progress and senesce earlier than female floral buds. This is due to the female requirement for more resources to prepare for reproduction than that of males (Hultine, Bush, West, & Ehleringer, 2007; McDowell, McDowell, Marshall, & Hultine, 2000; Pickering & Arthur, 2003). In a study conducted with one, four‐ and ten‐year‐old *Populus* × *canadensis* aimed at checking the expression of miRNA's during vegetative phase change*,* it was found that the change is evident in minor changes in leaf shape and internode length (Wang et al., 2011). Our experimental plants changed leaf shape from triangular form to round form over the study years, demonstrating their transition from juvenile to vegetative phase. It may be that during the phase change, females show more responsive growth patterns compared to males, in order to compensate for their reproductive requirements.

To sum up, elevated temperature was influential in delaying and forcing the bud formation and development. The effect of UV‐B diminished during the second growing season, but was again seen in bud‐removed individuals for bud break 2015. Males were more responsive compared to females, and the removal of invested resources (axillary buds) from the plants resulted in delay and forcing of autumn and spring bud phenology in males when compared to females. As the timing of bud break and bud set represents events in survival and growth, discernment of these mechanisms and their interactions with climatic variables is a key to understand the consequences of the projected climate change for *Populus* forests.

## AUTHORS CONTRIBUTION

As the corresponding author Unnikrishnan Sivadasan was responsible for the study conception and design, data acquisition, analysis and interpretation the study, and writing the manuscript. As my supervisors Riitta Julkunen Tiitto and Line Nybakken designed, directed, and coordinated the study providing conceptual and technical guidance for all aspects of the study. Chenhao Cao contributed to the data acquisition. Tendry Randriamanana contributed to the statistical analysis. Virpi Virjamo provided valuable comments in the research study. All authors contributed to the drafting of the manuscript to its final version of submission.
